# Impact of Temporal Visual Flicker on Spatial Contrast Sensitivity in Myopia

**DOI:** 10.3389/fnins.2021.710344

**Published:** 2021-08-05

**Authors:** Jie Ye, Pawan Sinha, Fang Hou, Xianghang He, Meixiao Shen, Fan Lu, Yilei Shao

**Affiliations:** ^1^School of Ophthalmology and Optometry, Wenzhou Medical University, Wenzhou, China; ^2^Department of Brain and Cognitive Sciences, Massachusetts Institute of Technology, Cambridge, MA, United States; ^3^Fuzhou Aier Eye Hospital, Fuzhou, China

**Keywords:** visual plasticity, myopia, spatial contrast sensitivity, temporal visual flicker, flicker adaptation

## Abstract

**Purpose:**

To investigate whether short-term exposure to high temporal frequency full-field flicker has an impact on spatial visual acuity in individuals with varying degrees of myopia.

**Methods:**

Thirty subjects (evenly divided between control and experimental groups) underwent a 5-min exposure to full-field flicker. The flicker rate was lower than critical flicker frequency (CFF) for the experimental group (12.5 Hz) and significantly higher than CFF for the controls (60 Hz). Spatial contrast sensitivity function (CSF) was measured before and immediately after flicker exposure. We examined whether the post flicker CSF parameters were different from the pre-exposure CSF values in either of the subject groups. Additionally, we examined the relationship between the amount of CSF change from pre to post timepoints and the degree of subjects’ myopia. The CSF parameters included peak frequency, peak sensitivity, bandwidth, truncation, and area under log CSF (AULCSF).

**Results:**

There was no significant difference of all five pre-exposure CSF parameters between the two groups at baseline (*P* = 0.333 ∼ 0.424). Experimental group subjects exhibited significant (*P* < 0.005) increases in peak sensitivity and AULCSF, when comparing post-exposure results to pre-exposure ones. Controls showed no such enhancements. Furthermore, the extent of these changes in the experimental group was correlated significantly with the participants’ refractive error (*P* = 0.005 and 0.018, respectively).

**Conclusion:**

Our data suggest that exposure to perceivable high-frequency flicker (but, not to supra-CFF frequencies) enhances important aspects of spatial contrast sensitivity, and these enhancements are correlated to the degree of myopia. This finding has implications for potential interventions for cases of modest myopia.

## Introduction

Myopia is one of the foremost causes of visual impairment around the world (Wong et al., 2014; Ohno-Matsui et al., 2016). Although the exact etiology of myopia is still unknown, genetic as well as environmental factors are implicated. One such environmental factor is exposure to visual flicker. High-frequency flicker has been found to suppress the axial elongation of animal eyes (Shih et al., 1997; Li et al., 2012). The secretion of compounds such as dopamine (DA) and crystallin proteins, are believed to be involved in linking flicker and myopia (Shih et al., 1997; Li et al., 2012).

From the perspective of neurophysiology, the influence of flicker on myopia is puzzling. The mammalian visual pathway is generally divided into two major streams of processing, parvocellular and magnocellular. The former has sensitivity to high spatial frequency stimuli while the latter is sensitive to high temporal frequencies (Burr and Ross, 1982; Livingstone and Hubel, 1987; Lee et al., 1990; Geisler et al., 2001). Given that the most evident impact of myopia is on spatial contrast sensitivity, especially in the high spatial frequency regime, the parvocellular pathway is believed to be more strongly involved in the genesis of myopia (Oen et al., 1994; Hashemi et al., 2012). The reduction of high spatial frequency details in the retinal image, especially during near-work with accommodation lag, would be expected to lead to axial elongation and further myopic development (Woodman et al., 2011; Hughes and Read, 2020). The magnocellular pathway’s involvement in myopia remains unclear. Probing the effect of temporal flicker, which is the purview of the magnocellular system, on myopia, can help reveal whether this pathway does in fact play a role in the condition.

Studies with non-human subjects have provided initial evidence for the influence of flicker on myopia. Given these results, it is important to explore how this factor impacts human vision; the results can have implications for our basic understanding of myopia as well as for the design of practical interventions for this condition. The objective of the current study was to investigate the effect of short-term flicker on human spatial visual acuity and, especially, its correlation with myopia.

## Materials and Methods

### Subjects

Thirty young adult subjects from Wenzhou Medical University were enrolled in the study and were randomly assigned to equal-sized control and experimental groups. All subjects had a comprehensive primary eye examination including measurements of the refractive error and best-corrected visual acuity (BCVA), slit-lamp examinations, axial length (AL, measured by IOL-master 500; IOLMaster, Carl Zeiss Meditec, Dublin, CA, United States), and so on. The BCVA was recorded as the logarithm of minimal angle resolution (LogMAR). The inclusion criteria were as follows: age between 20 and 30 years old, refractive error range from −8.0 diopter (D) to +0.5 D with astigmatism less than −1.5 D, and BCVA ≤ 0. None of the subjects had any systemic disease, ocular pathology, history of laser treatment, trauma, or eye surgery. All subjects who participated in this study signed consent forms and were treated in accordance with the tenets of the Declaration of Helsinki. This study was approved by the Ethics Committee of Wenzhou Medical University and all subjects were naïve as to the specific purpose of this experiment.

### Spatial Contrast Sensitivity Function Test

**FIGURE 1:**
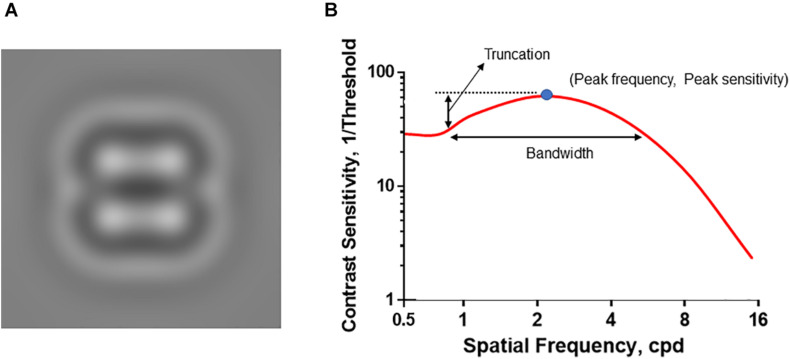
Introduction of qCSF visual stimuli and CSF curve. **(A)** The representative visual stimuli sample. The bandpass-filtered digits were used as the visual stimuli to measure the contrast sensitivity thresholds. **(B)** The CSF curve. Peak sensitivity, peak gain; Peak frequency, the spatial frequency corresponding to peak gain; Bandwidth, the function’s full-width at half peak sensitivity; Truncation, the distance between the peak sensitivity and plateau.

A custom-built quick contrast sensitivity function (qCSF) system with digit stimuli was used to test the spatial contrast sensitivity function (CSF). Details of qCSF software have been reported in our previous study (Zheng et al., 2018). Briefly, the qCSF software was run in MATLAB (MathWorks, Natick, MA, United States) on an Apple Mac mini-computer (Model No. A1347; Apple Inc., Cupertino, CA, United States) with a 60 Hz refresh rate and mean luminance of 91.2 cd/m^2^. The CSF test was conducted binocularly with the subject’s best refractive error correction. Contrast sensitivity thresholds for spatial frequencies ranging from 0.50 to 15.8 cycle per degree (cpd) were measured. The bandpass-filtered digits were used as the visual stimuli to measure the contrast sensitivity thresholds (Figure 1A). The center frequency of the filter was designed to be three cycles per object, with one-octave full bandwidth at half height (Zheng et al., 2018). Each digit visual stimulus duration was 133 ms, which reduced the likelihood of saccade execution during stimulus presentation and was suitable for the temporal integration (Breitmeyer and Ganz, 1977; Legge, 1978; Purves et al., 2001). Each experimental session comprised 100 trials of two consecutive qCSF runs (50 trials × 2 runs). The subjects responded with what digit they saw at each trial. The measurement duration of the CSF test was approximately 2–3 min. From the test results, we derived the peak sensitivity (the peak gain), peak frequency (the spatial frequency corresponding to peak gain), bandwidth (the function’s full width at half peak sensitivity), truncation (the distance between the peak sensitivity and plateau), and the area under log CSF (AULCSF) by CSF curve fitting (Figure 1B). The accuracy and repeatability of the qCSF system have been demonstrated in our previous paper (Zheng et al., 2019).

### Process

To have all subjects start from a comparable baseline, they were dark-adapted for a period of 10-min. The subsequent test process for the control and experimental groups differed in the following way: After dark adaptation, **control** subjects were assessed via qCSF system and then asked to view for a period of 5 min full-field flicker with a temporal frequency of 60 Hz square-wave with an equal duty cycle [which, by virtue of being significantly higher than human critical flicker frequency (CFF)], was perceived as a steady field. They were then retested using the qCSF system. The **experimental group** also underwent baseline CSF assessment after the 10-min dark adaptation. Subsequently, they viewed full-field flicker with a temporal frequency of 12.5 Hz square-wave with an equal duty cycle for 5-min. They were then retested using the qCSF system. The full-field flicker was presented as a Ganzfeld setup. A pair of safety-goggles with the transparent surface filling the entire visual field and lined with translucent vellum was used. A flickering light observed while wearing these goggles was experienced as a full-field flicker. The full-field flicker stimuli that the control and experimental subjects viewed were matched in their mean luminance (91.2 cd/m^2^). All subjects viewed the stimuli with best refractive error corrections by wearing glasses while positioned in a chin rest to avoid the movement of the position and distance from the stimuli (1.34 m). None of the subjects reported experiencing any discomfort during the session.

### Statistical Analysis

All data were analyzed with SPSS software (version 22.0; SPSS, Inc., Chicago, IL, United States). All continuous variables were calculated as means ± standard deviations (SDs). The refractive error was transformed in the form of the spherical equivalent comprising the spherical diopter plus half of the cylindrical diopter. Only the refractive error of the dominant eye was used for further comparison and correlation analysis. The dominant eye was determined by the Hole-in-card test (Seijas et al., 2007). The post alterations in the CSF parameters from baseline were analyzed. Paired *t*-tests were used to compare the differences between the two groups and the two measurement points (baseline and post). The repeated measures analysis of variance (ANOVA) was also used to compare the differences of five CSF parameters with the two-measurement points as the within-subjects variables and groups as between-subjects factors. The general estimating equation was used to adjust the influence of baseline CSF parameters on the comparisons of the post alteration of the CSF parameters between the two groups. Pearson and partial correlation were used to analyze the correlation between the alteration magnitudes and refractive error. The *P* < 0.05 was considered as the statistical significance.

## Results

### Basic Patients Characteristics

There were 15 subjects (Female: Male = 11:4) in the control group and 15 (Female: Male = 11:4) in the experimental group, respectively. Control and experimental groups did not differ significantly in age (25.8 ± 1.9 years vs. 26.2 ± 2.1 years, *P* = 0.327). There was no significant difference in refractive error between the control and experimental group (−5.86 ± 1.73 D vs. −6.01 ± 1.76 D, *P* = 0.413). The axial length of control group was similar with experimental group (25.32 ± 0.62 mm vs. 25.30 ± 0.63 mm, *P* = 0.442).

### Differences in CSF Alteration in the Two Groups

There was no significant difference between control and experimental groups for any of the five CSF parameters at baseline (*P* = 0.333 ∼ 0.424, Table 1). However, post-flicker exposure CSF measurements revealed notable differences between the groups.

**TABLE 1:** Summary of mean qCSF parameters at baseline, post alteration from baseline in two groups.

	Peak sensitivity	Peak frequency	Bandwidth	Truncation	AULCSF
**Baseline**					
Control group (*N* = 15)	54.882 ± 11.360	1.317 ± 0.411	3.107 ± 0.461	0.139 ± 0.044	15.667 ± 6.920
Experimental group (*N* = 15)	53.087 ± 11.230	1.347 ± 0.425	3.071 ± 0.455	0.136 ± 0.033	15.001 ± 6.213
*P*	0.333	0.424	0.415	0.421	0.392
**Post Alteration from Baseline**					
Control group (*N* = 15)	1.552 ± 5.570	−0.111 ± 0.339	0.134 ± 0.423	−0.003 ± 0.045	0.685 ± 2.452
Experimental group (*N* = 15)	6.374 ± 7.241	−0.029 ± 0.446	0.150 ± 0.459	−0.011 ± 0.044	3.588 ± 3.595
*P*	**0.025 (0.017)**	0.287 (0.278)	0.462 (0.461)	0.321 (0.314)	**0.016 (0.010)**

*AULCSF, the area under log CSF; *P-*value between the control and experimental groups. Values in parentheses were the *p-*values after the adjustment for the corresponding data at baseline by the general estimating equation. The bold values just means the P-value less than 0.05.*

**FIGURE 2:**
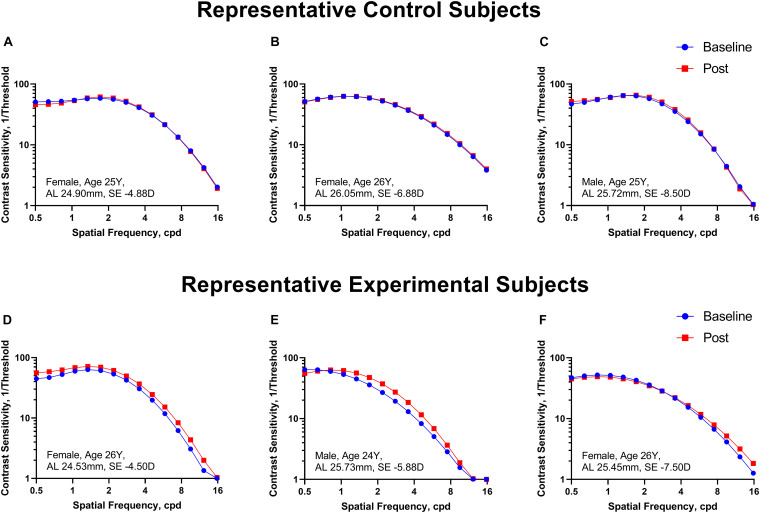
The CSF curves in six representative cases from the control and experimental groups. **(A–C)** Control group. **(D–F)** Experimental group. Cpd, cycle per degree.

**FIGURE 3:**
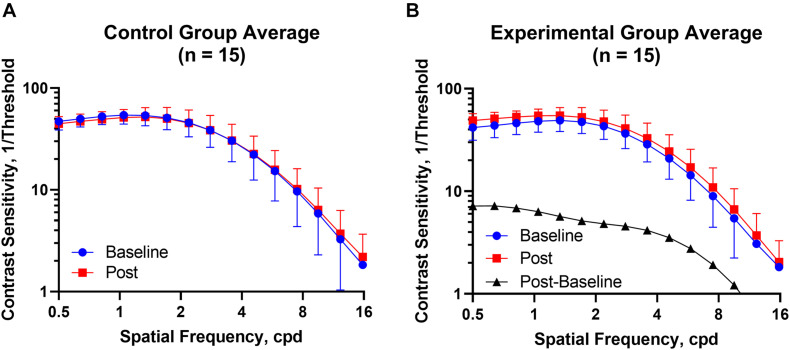
The CSF curves in the control and experimental groups with average CSF and standard deviation value. **(A)** Control group. **(B)** Experimental group. Cpd, cycle per degree.

**FIGURE 4:**
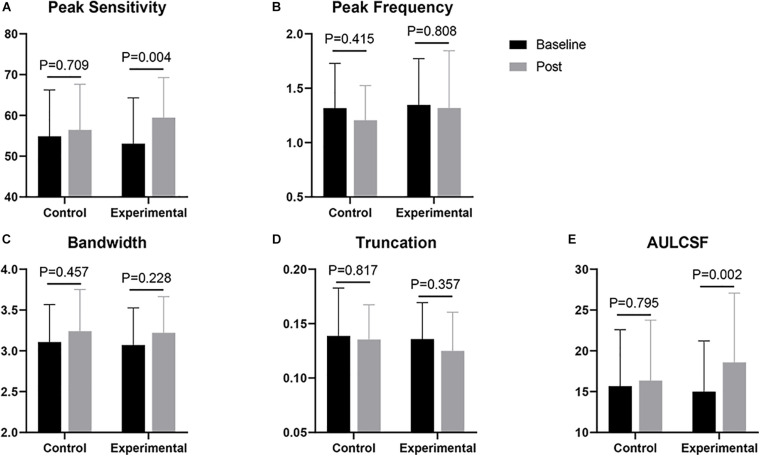
Comparisons of CSF parameters between baseline and post. **(A)** Peak sensitivity; **(B)** Peak frequency; **(C)** Bandwidth; **(D)** Truncation; **(E)** AULCSF. The *p*-value shown in each panel corresponds to the result of a *t*-test.

**FIGURE 5:**
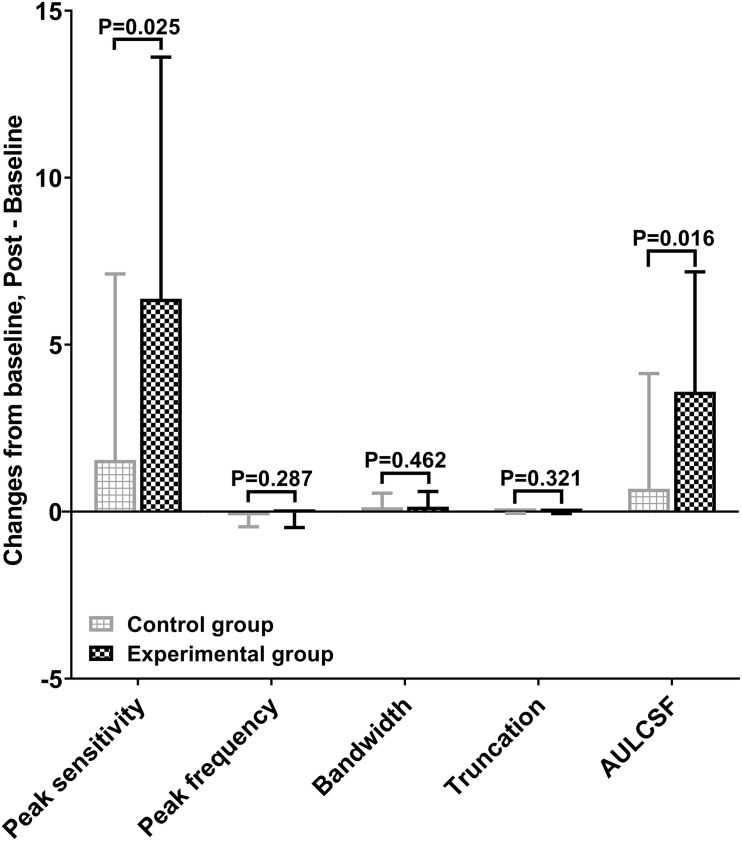
Mean changes of post CSF parameters from baseline in control and experimental group.

Figure 2 shows the CSF curves of six representative subjects (three control and the other three experimental) at baseline and post-flicker. Figure 3 shows the average CSF curves in the control and experimental groups with their standard deviation values. Control group participants exhibited no significant changes in their CSF parameters from before to after flicker exposure (Figure 4). However, significant changes of peak sensitivity and AULCSF were evident in the data from the experimental group (Figures 4A,E). For the post five CSF parameters, a repeated measures ANOVA showed that the flicker exposures have a significant effect in peak sensitivity [*F*(1,28) = 11.291, *P* = 0.002] and AULCSF [*F*(1,28) = 11.022, *P* = 0.003]. There was a significant interaction between group and flicker exposure in peak sensitivity [*F*(1,28) = 4.180, *P* = 0.049] and AULCSF [*F*(1,28) = 5.088, *P* = 0.032]. Figure 5 plots the data in terms of changes from the baseline, and highlights the significant differences of two parameters (peak sensitivity: Control group 1.552 ± 5.570 vs. Experimental group 6.374 ± 7.241, *P* = 0.025; AULCSF: Control group 0.685 ± 2.452 vs. Experimental group 3.588 ± 3.595, *P* = 0.016; Figure 5, Paired *t*-tests).

### Correlation Between the CSF Alteration and Refractive Error

**FIGURE 6:**
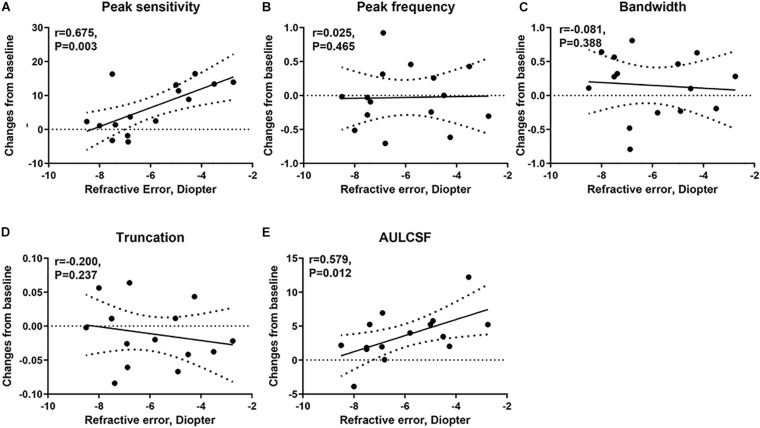
The correlation of the changes in CSF parameters from baseline and refractive error in the experimental group. **(A)** The correlation of the changes in peak sensitivity from baseline and refractive error. **(B)** The correlation of the changes in peak frequency from baseline and refractive error. **(C)** The correlation of the changes in bandwidth from baseline and refractive error. **(D)** The correlation of the changes in truncation from baseline and refractive error. **(E)** The correlation of the changes in AULCSF from baseline and refractive error.

We computed the correlation between the post-flicker alteration of CSF parameters and magnitude of refractive error in the experimental group. We found peak sensitivity (*r* = 0.675, *P* = 0.003) and AULCSF (*r* = 0.579, *P* = 0.012) to be correlated with the refractive error (Figure 6 and Table 2). These significant correlations still existed after adjusting the corresponding baseline CSF parameters (Table 2) in the experimental group. There was no statistically significant association of the refractive error with post-flicker alteration of peak frequency, bandwidth, and truncation in the experimental group (*r* = −0.200 ∼ 0.025, *P* = 0.237 ∼ 0.465, Figure 6 and Table 2). In the control group, there were no significant correlations between CSF parameters alteration and refractive error (*r* = −0.277 ∼ 0.444, *P* = 0.097 ∼ 0.845).

**TABLE 2:** The correlation between the post alteration CSF parameters from baseline and the refractive error in the experimental group.

	*r*	*P*	P*
Peak sensitivity	0.675	**0.003**	**0.005**
Peak frequency	0.025	0.465	0.358
Bandwidth	–0.081	0.388	0.360
Truncation	–0.200	0.237	0.069
AULCSF	0.579	**0.012**	**0.018**

*The *P*-value of the correlation between the post alteration CSF parameters from baseline and the refractive error after adjusting the corresponding baseline CSF parameters. The bold values just means the P-value less than 0.05.

## Discussion

We evaluated the impact on spatial contrast sensitivity of short-term exposure to perceivable high-frequency flicker, in myopic patients. Our data revealed that the peak sensitivity and AULCSF increased significantly after exposure to merely 5 min of perceivable high-frequency flicker, but not to flicker with a frequency greater than CFF. Furthermore, since the refractive error increased with negative numbers, the magnitude of increase in these parameter values was significantly negatively correlated to the severity of myopia with greater changes associated with less degree of myopia.

Previous work has reported that flicker adaptation raises spatial frequency of “coarse” test gratings, but does not yield any improvements with finer gratings (Kaneko et al., 2015). However, Arnold et al. (2016) have reported improvements in the ability to see fine facial details after flicker exposure. In the present study, we used digit stimuli (as a finer test method) (Zheng et al., 2019) to detect the effect of flicker exposure on high spatial frequency perception, and the results demonstrate significant improvements. It is worth noting that although the CSF value was lower with our qCSF system than traditional method, it would be similar to traditional value when converted into the root mean square contrast, as has been verified in our previous paper (Zheng et al., 2019).

The rapid alteration of the CSF curve following brief flicker exposure indicates plasticity of the mechanisms underlying fine spatial acuity in adults. What is notable is that the manipulation (temporal flicker) is designed to impact the magnocellular pathway, while the consequence (changes in fine spatial contrast sensitivity) is associated with the parvocellular pathway. While the precise mechanisms by which this cross-pathway effect is obtained are yet unclear, we speculate that the shape of the CSF curve, especially the peak spatial frequency and AULCSF, are jointly governed by the parvocellular and magnocellular pathways rather than by the former alone (Kaneko et al., 2015). Short-term perceivable high-frequency flicker may lead to adaptation of the magnocellular pathway and a corresponding reduction in its contribution to spatial CSF relative to that of the parvocellular pathway. This parvo-favoring imbalance may lead to improving the acuity of spatial vision (Kitterle and Beard, 1983; Zhuang et al., 2015). Prior work suggests that the interaction between the two pathways may take the form of suppression, with the magnocellular pathway exerting a suppressive influence on the parvocellular pathway under normal photopic conditions (Smith, 1971; Cass and Alais, 2006). Adaptation of the magnocellular pathway by the short-term perceivable high-frequency flicker may lift some of this suppression of the parvocellular pathway, with a consequent improvement of high-spatial frequency visual perception.

One important doubt for us now is whether 0.5 cpd was low enough to be mainly within the purview of the magnocellular pathway, as the qCSF software in the current study measured CSF from 0.5 to 15.8 cpd. Previous study showed that after adaption to the flicker could knock out the transient channels, and then (by implication) reduce the sensitivity to low spatial frequencies (Kaneko et al., 2015), which might be different from the Figure 3 curve in our study. Kaneko et al. (2015) considered the 3 cpd as the cutoff spatial frequency for the transient and sustained channels. While, past work suggested that in macaque monkeys, 2 cpd was still mainly modulated by parvocellular pathway (Merigan et al., 1991). In our previous study, we already found that the congenital cataract children with serious impaired spatial visual function but not temporal visual function were with peak sensitivity at around and even less than 0.5 cpd after surgery (Ye et al., 2021). Hence, the range of the spatial frequencies we measured in the current study might still be mainly subserved by the parvocellular pathway. We acknowledged the puzzlement regarding the observed differences in the contrast sensitivity at low and high spatial frequencies after flicker experience. We believe that future studies with larger sample sizes are needed to pursue this potentially interesting finding.

In the current study, subjects were asked to gaze at the full-field flicker stimulus without any specific accommodation stimuli. The data revealed that this effect was flicker-specific and not simply the outcome of prolonged light stimulation (Riddell, 1935; Ginsburg, 1966) since it was found to occur when subjects were exposed to a perceivable flicker but not a seemingly steady light (Riddell, 1935; Ginsburg, 1966). Additionally, the flicker effect was not related to the spatial extent of the stimuli (Zhuang et al., 2015). The flicker stimuli were much easier than pattern stimuli to be recognized (Kitterle and Beard, 1983). These results have implications for understanding spatial acuity outcomes following treatment for congenital cataracts. Severe congenital cataract patients have access to full-field flicker despite being unable to resolve any spatial forms. This temporal experience may facilitate the development of spatial function, and thus potentially account for the observed spatial acuity outcomes in such individuals (Kalia et al., 2014).

The mechanistic underpinnings for explaining the association between high-frequency flicker and myopia are yet unclear. At the same time, the perceptual learning produces roles in myopia is under popular discussion. It had been reported the perceptual learning with longer duration and even for several days would improve the visual function in myopia by improving neural procession (Camilleri et al., 2014, 2016; Yan et al., 2015). The flicker effect in our current study might not produce improvements comparable with those obtained with perceptual learning on visual function due to different mechanisms or short flicker duration. We still need to consider the underpinning of the flicker effect on myopia. In non-human animal models, it has been reported that deprivation and defocus induced myopia is inhibited by flicker of frequency 6 Hz or above (Gottlieb et al., 1991; Li et al., 2012). This may be because high-frequency flicker causes a hypermetropic shift, leads to hyperactivity of ganglion cells, and stimulates the release of DA from the retina, thereby reducing receptive field sizes, which may play a role in CSF, and even suppress myopic development (Shih et al., 1997; Crewther et al., 2006; Zhang et al., 2011). There may also be flicker-induced arrest of axial elongation and a corresponding reduction in myopic progression. Myopia has previously been shown to be strongly associated with axial elongation (Schwahn and Schaeffel, 1997; Read et al., 2010) perhaps as a consequence of the blurry retinal images with higher internal noise, especially during the near-work with accommodation lag (Yan et al., 2015). With the perceivable high-frequency flicker role on the improved spatial visual acuity, even might be with reduced internal noise, we speculated the effect of the axial elongation would be reduced. Interestingly, it has been reported that children with form-deprivation myopia induced by severe congenital cataracts do not exhibit large axial elongation, compared to age-matched normally sighted children to some extent (Kun et al., 2018). This axial elongation may be prevented by the access the children have to full-field flicker without the accompanying spatial signal that may, on its own, induce elongation. Although this potential link between flicker and myopia, through the intermediary of axial length deserves further exploration, its use as an explanatory mechanism for our results is probably potentially meaningful, given the rapidity with which flicker is found to alter spatial function.

Our results reveal that the effect of flicker on myopia is related to the degree of myopia. The higher the degree of myopia, the less the flicker effect on spatial visual function. What might account for this negative correlation? We offer a tentative hypothesis. High myopia might impact parvocellular and magnocellular pathways. The retinal nerve fiber layer (RNFL), the origination of the parvocellular and magnocellular pathways, is reported to be thinner in high myopia, especially in the peripheral area (Wang et al., 2018; Lee et al., 2019). This is especially evident in the RNFL of the magnocellular pathway in the peripheral retina. Hence, the significant decrease of RNFL thickness in high myopia might lead to a reduced sensitivity of the magnocellular pathway to flicker stimuli, which in turn reduces the pathway’s impact on spatial function.

We note some limitations in our current study. Our sample size is modest, especially from the perspective of assessing correlations between CSF parameters and refractive error. Although previous psychophysical and neurophysiological studies have demonstrated the usefulness of small samples (Anderson and Vingrys, 2001), the current results need to be verified with larger cohort sizes for us to be able to repose confidence in their replicability. We have also not systematically varied the duration of the flicker exposure to probe how it modulates the impact on spatial CSF, though it had been reported that the contrast sensitivity alteration was unrelated to a further longer duration (Kitterle and Beard, 1983). The persistence of the results also needs further research to determine how long-lasting the effects are and how they are influenced by the duration of flicker exposure. Finally, future work can derive correlation values not just between dominant eye refractive error and CSF parameter change (as we have done here), but also non-dominant eye error, with both binocular and monocular CSF values. In this context, it is worth noting that the flicker effect is consistent between monocular and binocular presentations (Zhuang and Shevell, 2015).

In summary, we have found that perceivable high-frequency flicker enhances the acuity of spatial vision in myopic individuals, and this enhancement is negatively correlated with the degree of myopia. The mechanisms underlying this influence are not definitively established yet, but may involve reduced suppressive interactions between magnocellular and parvocellular pathways and, over longer timescales, arrest of axial elongation by flicker. If these results are corroborated in future studies with more participants, we expect them to have important implications for the design of interventions of cases of moderate myopia.

## Data Availability Statement

The raw data supporting the conclusions of this article will be made available by the authors, without undue reservation.

## Ethics Statement

The studies involving human participants were reviewed and approved by the Ethics Committee of Wenzhou Medical University. The patients/participants provided their written informed consent to participate in this study. Written informed consent was obtained from the individual(s) for the publication of any potentially identifiable images or data included in this article.

## Author Contributions

JY, PS, and FL contributed to the conception and design of the study. JY and FH determined the experimental methods. JY performed the experiments. JY and XH analyzed and interpreted the data. JY, MS, and YS wrote and modified the manuscript. All authors contributed to manuscript revision, read, and approved the submitted version.

## Conflict of Interest

The authors declare that the research was conducted in the absence of any commercial or financial relationships that could be construed as a potential conflict of interest.

## Publisher’s Note

All claims expressed in this article are solely those of the authors and do not necessarily represent those of their affiliated organizations, or those of the publisher, the editors and the reviewers. Any product that may be evaluated in this article, or claim that may be made by its manufacturer, is not guaranteed or endorsed by the publisher.
